# Evaluation of Two Major *Rhodiola* Species and the Systemic Changing Characteristics of Metabolites of *Rhodiola crenulata* in Different Altitudes by Chemical Methods Combined with UPLC-QqQ-MS-Based Metabolomics

**DOI:** 10.3390/molecules25184062

**Published:** 2020-09-05

**Authors:** Xueda Dong, Yiwen Guo, Chuan Xiong, Liwei Sun

**Affiliations:** National Engineering Laboratory for Tree Breeding, College of Biological Sciences and Biotechnology, Beijing Forestry University, Beijing 100083, China; hbxd0327@163.com (X.D.); m17611583050@163.com (Y.G.); xxiongchuan@163.com (C.X.)

**Keywords:** *Rhodiola*, species, elevation, UPLC-QqQ-MS-based metabolomics, biomarker, differential metabolites, phytochemicals

## Abstract

*Rhodiola* species have a long history of use in traditional medicine in Asian and European countries and have been considered to possess resistance to the challenges presented by extreme altitudes. However, the influence of different *Rhodiola* species on quality is unclear, as well as the influence of altitude on phytochemicals. In this study, the phenolic components and antioxidant abilities of two major *Rhodiola* species are compared, namely *Rhodiola* *crenulata* and *Rhodiola rosea*, and the metabolomes of *Rhodiola* *crenulata* from two representative elevations of 2907 and 5116 m are analyzed using a UPLC-QqQ-MS-based metabolomics approach. The results show that the phenolic components and antioxidant activities of *Rhodiola* *crenulata* are higher than those of *Rhodiola rosea*, and that these effects in the two species are positively correlated with elevation. Here, 408 metabolites are identified, of which 178 differential metabolites (128 upregulated versus 50 downregulated) and 19 biomarkers are determined in *Rhodiola crenulata*. Further analysis of these differential metabolites showed a significant upregulation of flavonoids, featuring glucosides, the enhancement of the phenylpropanoid pathway, and the downregulation of hydrolyzed tannins in *Rhodiola crenulata* as elevation increased. Besides, the amino acids of differential metabolites were all upregulated as the altitude increased. Our results contribute to further exploring the *Rhodiola* species and providing new insights into the *Rhodiola crenulata* phytochemical response to elevation.

## 1. Introduction

The *Rhodiola* species is a species of alpine herb. The rhizome of this species has been used in traditional medicine in Asian and European countries to improve overall health. There are approximately 90 species worldwide, and 73 species are found in China. *Rhodiola crenulata* and *Rhodiola rosea* are the main medicinal species [1,2]. According to Flora of China [3,4], *Rhodiola crenulata* is mainly distributed in Tibet (also named Xizang), China, and *Rhodiola rosea* exists in Jilin (not found in Tibet), China. However, the qualities of these two species have not yet been evaluated. *Rhodiola* grows on alpine grasslands, valley rocks, or glaciers at an altitude range of 1800–5600 m [3,4] and can adapt to extremely high altitude adversities, including a low temperature, hypoxia, intensive ultraviolet radiation, huge diurnal temperature differences, etc. Studies have shown that *Rhodiola* contains many phytochemicals, such as flavonoids, phenylpropanoids, tannins, amino acids, alkaloids, organic acids, and volatile oils, etc. [5,6,7]. Furthermore, modern research has shown that the comprehensive nourishing effects of the *Rhodiola* species are largely attributed to its phytochemicals [6], which exert anti-hypoxic [8], anti-viral [9], immune regulatory [10], anti-tumor [11], anti-fatigue [12], anti-depressive [2], and improvement of learning and memory [13] effects.

Strong links exist between phytochemicals and environmental stresses in plants. Plant flavonoids are the major molecular defensive substances [14] and are biosynthetically associated with phenylpropanoids and tannins, and these substances are collectively known as plant phenolics. Reports have shown that the antioxidant abilities and contents of phenolics of medicines are correlated with altitude [15,16,17]. Ultraviolet radiation and the low temperature conditions promote flavonoid biosynthesis, thus leading to the accumulation of flavonoids and glycosides in plants [18,19]. Tannins protect plants against biotic stresses [20]. Structurally, phenolic hydroxyl groups possess strong reducibility, thus conferring plant phenolics vital protection against oxidative stress induced by environmental adversities [21]. Alkaloids are known to be greatly affected by ultraviolet (UV) radiation and temperature [22,23]. Organic acids are fundamental intermediate products in the tricarboxylic acid cycle (TCA), pertaining to the aerobic respiratory metabolic pathway in plants [24]. Amino acids play a wide variety of physiological roles in plants, including promoting growth and development, improving drought [25] and salt tolerance [26], and offering phenylalanine as an intermediate in the biosynthesis of most plant phenolics [27]. Moreover, a report has shown that the content of salidroside in *Rhodiola sachalinensis* is associated with altitude [28], suggesting that altitude might be an important factor that affects phytochemicals in *Rhodiola*. However, there is currently a lack of documentation regarding the systemic changing characteristics of phytochemicals of *Rhodiola* caused by elevation, thus demanding prompt investigation.

In the present study, an ultra-high-performance liquid chromatography coupled to triple quadrupole mass spectrometry (UPLC-QqQ-MS)-based metabolomics approach is used. This approach is more sensitive and accurate for detecting metabolites [29] and used here to analyze the types and relative contents of phytochemicals in *Rhodiola crenulata* at different elevations, including flavonoids, hydrolyzed tannins, phenylpropanoids, amino acids, alkaloids, and organic acids. Using chemical methods, we also evaluate the qualities of two major medicinal *Rhodiola* species, namely *Rhodiola crenulata and Rhodiola rosea*, grown in different region-altitude ranges (*Rhodiola crenulata* in Tibet, China, at altitudes of 2907 and 5116 m; *Rhodiola rosea* in Jilin, China, at altitudes of 1950 and 2248 m).

## 2. Results and Discussion

### 2.1. Evaluation Qualities of Two Major Medicinal Rhodiola Species Growing in Different Region-Altitude Ranges

To evaluate the qualities of *Rhodiola* species distributed at different region-altitude ranges, the characteristics of the phenolic components and antioxidant abilities of two famous medicinal *Rhodiola* species were first investigated, namely *Rhodiola crenulata* from Tibet, China (SJL, altitude of 2907 m; SN, altitude of 5116 m) and *Rhodiola rosea* from Jilin, China (JL1, altitude of 1950 m; JL2, altitude of 2248 m).

**Figure 1:**
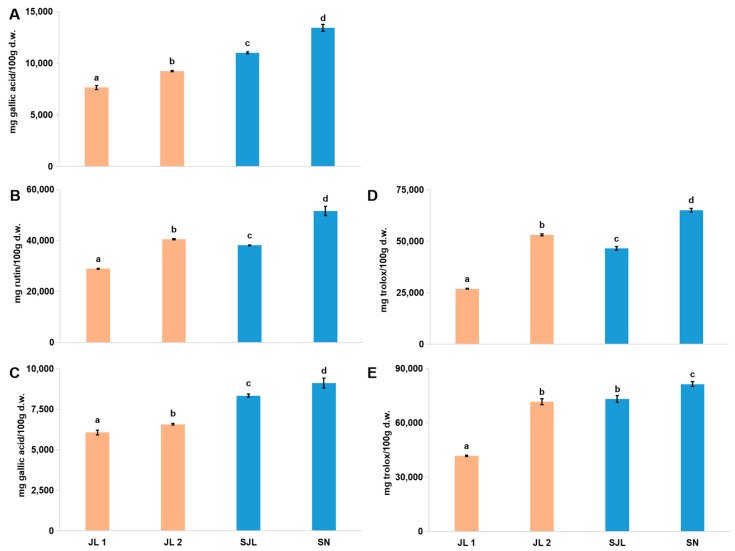
Characteristics of the phenolic components and antioxidant abilities of *Rhodiola* (JL1, JL2, SJL, and SN). (**A**): Total phenols; (**B**): Total flavonoids; (**C**): Total tannins; (**D**): DPPH antioxidant ability; (**E**): ABTS antioxidant ability. Gallic acid was used as a standard for the total phenols and total tannins. Rutin was used as a standard for the total flavonoids. Trolox was used as a standard for the DPPH and ABTS antioxidant abilities. Results are presented as the mean ± SD of three independent experiments (*n* = 3) and are expressed as mg standard per 100 g d.w. of plant material. In each column, different letters (a, b, c, d) mean significant differences between two groups (*p* < 0.05) found via a T-test. Note: *Rhodiola* samples are presented according to the ascending order of altitude from left to right.

As shown in Figure 1, the contents of total phenols, flavonoids, and tannins and the DPPH and ABTS antioxidant abilities were characterized in a rising pattern with an ascending order of altitude, i.e., JL1 < JL2 < SJL< SN, and significant differences were found between each sample. However, exceptions were found for the total flavonoids and DPPH (2,2-diphenyl-1-picrylhydrazyl) of JL2, which were higher than those of SJL, and no significance for ABTS + (2,2′-azino-bis (3-ethylbenzothiazoline-6-sulphonic acid) was found between JL2 and SJL. In general, these results indicate that the DPPH and ABTS scavenging abilities are related to the phenolic components, especially the total flavonoids. However, it should be noted that the sudden rise of total flavonoids in JL2 may be attributed to the strong response of this species to an increase in altitude.

Furthermore, although exceptions were found, the above order indicates that SJL and SN of *Rhodiola crenulata* in Tibet, China, are significantly different from JL1 and JL2 of *Rhodiola rosea* in Jilin, China, further exhibiting notable correlations for the phenolic components and antioxidant abilities with an increasing altitude, both between and within these two species.

Therefore, a conclusion can be drawn that the phenolic components and antioxidant abilities of *Rhodiola crenulata* from Tibet, China, are superior to those of *Rhodiola rosea* from Jilin, China. This conclusion is in accordance with the documentation in the Chinese Pharmacopoeia, where *Rhodiola crenulata* is said to be the authentic *Rhodiola* medicine [1].

Our findings are consistent with many reports. Lu et al. (2019) documented that *Lycium barbarum* L. fruit possesses better quality when grown closer to its natural growth location [30]. Farhat et al. (2013) showed that changes in habitat and altitude lead to significant variation in the phenolic contents and antioxidant activity of *Salvia verbenaca* L. [31]. Therefore, our conclusion for *Rhodiola crenulata* from Tibet, China, being superior to *Rhodiola rosea* from Jilin, China, may be an indication of Tibet in southwestern China being the authentic region for *Rhodiola* growth compared to Jilin in northeastern China. Moreover, the fact that the altitude in Tibet, China, is higher than that in Jilin, China, is consistent with our finding that the phenolic components and antioxidant abilities, both between and within the two species, display a significant correlation with altitude, suggesting that altitude instead of region might be the influential environmental factor regarding the chemical components and antioxidant ability for *Rhodiola*.

Our findings are in agreement with previous evidence regarding the enhancement of phenolic components and antioxidant capacities with an increased altitude in herbs [15,16,17]. Therefore, samples of *Rhodiola crenulata* from Tibet, China, were collected from two different altitudes of 2907 and 5116 m (SJL and SN), and the chemical components were analyzed.

### 2.2. Influences of Altitude on Chemical Compositions of Rhodiola crenulata 

As presented above, tremendous differences between both the total contents of the phenolic components and antioxidant activities between SJL and SN exist (Figure 1), suggesting that a wide range of chemical components in *Rhodiola crenulata* could be influenced by elevation. However, it is a fact that spectrophotometric methods merely probe the total contents of certain components, and therefore they cannot accurately depict subtle changes in individual compounds. To systematically characterize and gain more insight into chemical changes influenced by high altitudes, a new UPLC-QqQ-MS-based metabolomics approach was employed to analyze metabolites in two *Rhodiola crenulata* samples gathered from two representative high elevations at 2907 and 5116 m (i.e., SJL and SN).

**Figure 2:**
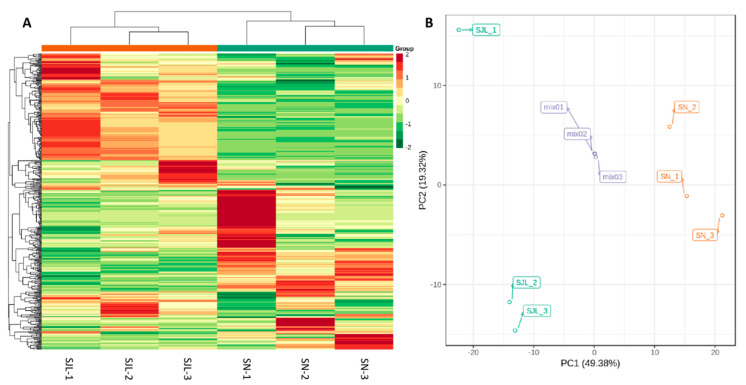
Heat map and principal component analysis (PCA) plots of samples from the two *Rhodiola crenulata* sample locations with three biological repeats for each sample. (**A**): Heat map; (**B**): Two-dimensional (2D) scatter plot of the PCA for SJL, SN, and the quality control (QC) sample. The QC was a mixture of SJL and SN.

Information on the metabolites analysis is listed in Table S1. Altogether, 408 metabolites were identified and then presented as a heat map (Figure 2A), indicating that the relative contents of the corresponding metabolites are similar between three biological repeats of the same sample, but strikingly different between the two different *Rhodiola crenulata* sample (SJL and SN).

The PCA plots for SJL, SN, and the quality control (QC) sample in Figure 2B show that obvious variations exist between SJL and SN, as presented by a 49.38% difference in PC1 and 19.32% difference in PC2, which is indicative of altitude exerting evident impacts on the compositions of *Rhodiola crenulata*.

### 2.3. Differential Metabolites for Rhodiola crenulata in Different Elevations 

**Figure 3:**
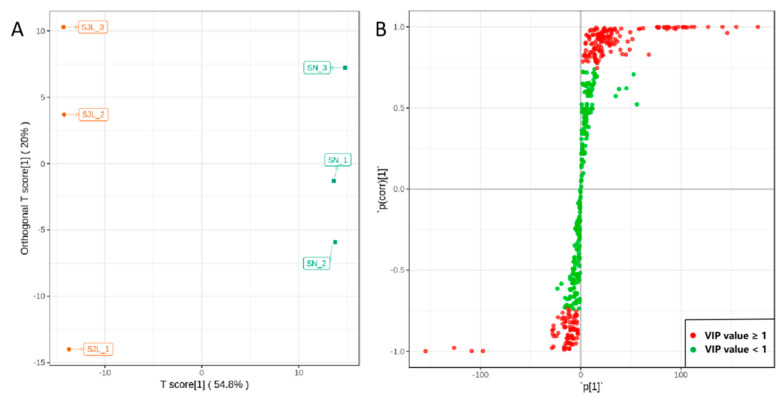
Orthogonal projections to latent structures discriminant analysis (OPLS-DA). (**A**): Score scatter plots of the OPLS-DA model for SJL vs. SN; (**B**): S-plot of the OPLS-DA model for SJL vs. SN, red dots denote metabolites with VIP value ≥ 1, while green dots mean metabolites with VIP value < 1.

The comparison of metabolite influence for SJL and SN by the orthogonal projections to latent structures discriminant analysis (OPLS-DA) model demonstrated that the model was meaningful and that the differential metabolites screened by the variable importance in project (VIP) value analysis are valid (Figure 3). Figure 3A shows that SJL and SN are clearly separated, as presented by the gathering of the SJL triplicates on the left, while SN on the right demonstrates visual changes in the metabolites between SJL and SN. Subsequently, the model was applied to build an S-plot (Figure 3B), in which individual metabolites are projected as dots, and metabolites that are far away from the origin line effectively distinguish between SJL and SN.

**Figure 4:**
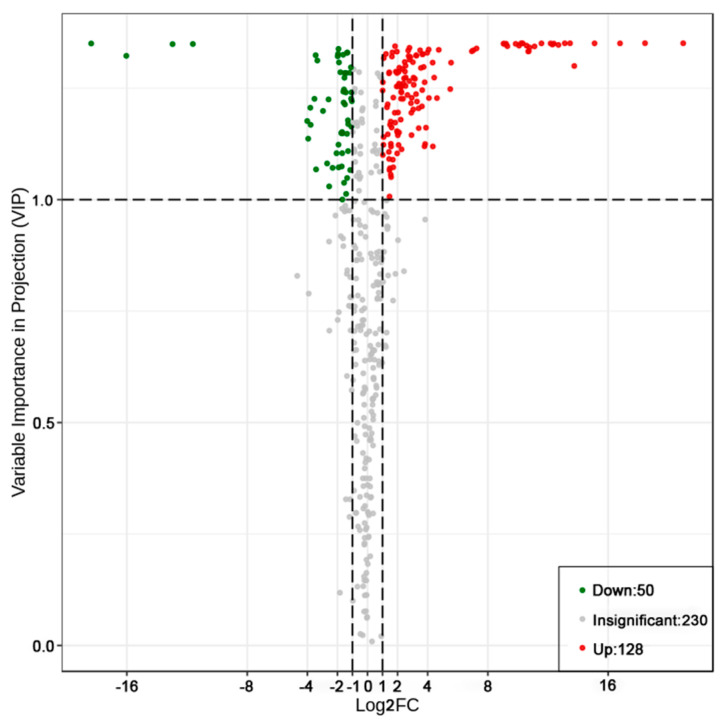
Differential metabolite analyses for SJL vs. SN with the criteria setting of variable importance in project (VIP) value ≥1 and FC ≥2 or ≤0.5, plotted by a volcano map. Red and green dots denote upregulated and downregulated differential metabolites, respectively; gray dots represent non-differential metabolites. Upregulation denotes that the content of a metabolite in SN exhibited a significant increase when compared with that in SJL. Conversely, downregulation indicates that the content of a metabolite in SN is significantly decreased when compared with the corresponding one in SJL.

Next, the 408 identified metabolites were used to screen differential metabolites based on the criteria of simultaneously meeting VIP ≥1 and FC ≥2 or ≤0.5, which was visualized as a volcano map (Figure 4). As Figure 4 shows, 178 metabolites, including 128 up- and 50 downregulated metabolites, were found to be significant and recognized as differential metabolites for describing the influence of altitude on *Rhodiola crenulata* (also see Table S2).

**Figure 5:**
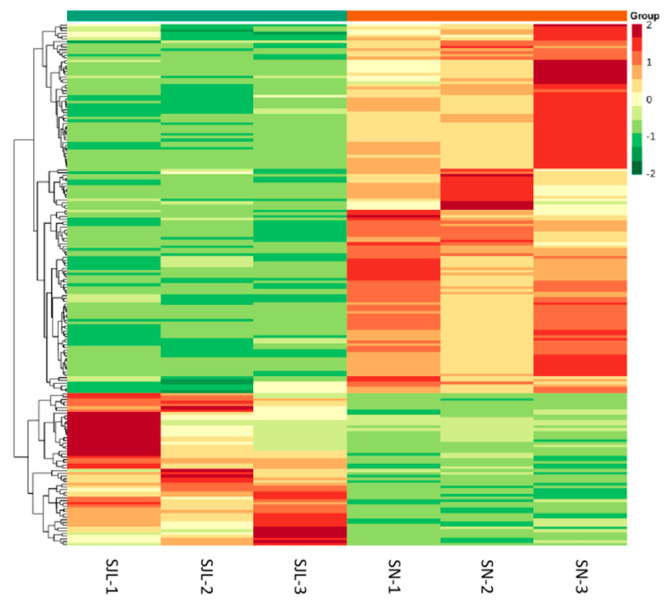
A heatmap for the relative variation of metabolites between SJL and SN. The color-coding from red to green indicates abundances from high to low, respectively.

The recognized differential metabolites between SJL and SN were further subjected to heatmap analysis to picture their relative contents (Figure 5). As shown in Figure 5, SJL sections composed of red and green color-coding are clearly distinguishable from those of SN, demonstrating significant differences in the relative contents of differential metabolites between SJL and SN.

### 2.4. Characterization of Biomarkers and Six Categories of Common Differential Metabolites

#### 2.4.1. Biomarkers

For the differential metabolites, nineteen that contributed greatly to the distinction between SJL and SN were first investigated (Table 1). Among these 19 metabolites, 16 of them were found in SN but not in SJL, denoting that the production of these 16 metabolites is a consequence of the elevation increasing from 2907 to 5116 m. Additionally, three existed only in SJL but not in SN, demonstrating that these three were strongly tied to an elevation at approximately 3000 m. Therefore, these 19 metabolites could be used as biomarkers to differentiate between *Rhodiola crenulata* grown at the two different elevations considered here.

Among the 16 only found in SN, six belong to flavonoids, covering a proportion of 37.5% of these 16. There is evidence that intensive UV radiation leads to the production of flavonoids in plants, which function as effective protectants against UV radiation, as well as low temperatures [18,32,33]. Based on the above documents and the common sense that an elevated altitude influences the environmental UV radiation and temperature, speculation can be made that these six flavonoids may be greatly responsible for the adaptation of *Rhodiola crenulata* to increased altitude-related stress, particularly for increased UV radiation, as well as a decrease in temperature, and they might be used as an index to evaluate the resistance of *Rhodiola crenulata* in terms of coping with altitude stresses.

#### 2.4.2. Six Categories of Common Differential Metabolites

Previous studies have documented that the major bioactive components of *Rhodiola* include flavonoids, hydrolyzed tannins (namely gallic acid derivatives), phenylpropanoids, amino acids, alkaloids, and organic acids [6,7]. Therefore, changes in these six major categories were subsequently described in detail. According to the criteria of simultaneously meeting a FC ≥2 or ≤0.5 and VIP value ≥1 (Table S2), 121 out of the 178 differential metabolites and excluding above biomarkers, including 50 flavonoids and flavonoid derivatives, 16 gallic acid derivatives, 26 phenylpropanoids, 14 amino acids, 8 alkaloids, and 7 organic acids between SJL and SN were named as common differential metabolites (CDMs) and subjected to further analysis.

##### Flavonoids

Fifty CDMs were identified as flavonoids, with 43 up- and 7 downregulated flavonoids, where, based on their corresponding aglycone, 38 were further classified into eight major subcategories with a large proportion of 76.00% existing in the total 50 flavonoid CDMs. These eight were kaempferol and its derivatives (12 CDMs), luteolin and its derivatives (8 CDMs), catechin and its derivatives (4 CDMs), apigenin and its derivatives (4 CDMs), quercetin and its derivatives (4 CDMs), herbacetin and its derivatives (2 CDMs), hesperetin and its derivatives (2 CDMs), and diosmetin and its derivatives (2 CDMs).

When combining the six upregulated flavonoids biomarkers (Table 1), 49 upregulated flavonoids versus 7 downregulated flavonoids were determined among 93 of the recognized flavonoids (also including 37 non-differential metabolites) in *Rhodiola crenulata*. This features a 52.69% upregulation of flavonoids, while there was a downregulation of only 7.53%, significantly indicating that *Rhodiola crenulata* synthesizes flavonoids more greatly as the growth elevation is increased.

**Figure 6:**
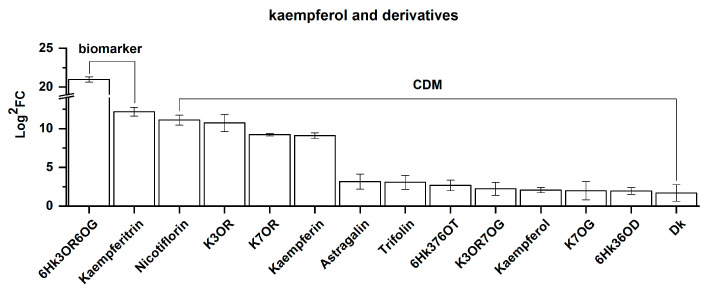
Comparison of fourteen differential metabolites of kaempferol and kaempferol derivatives between SJL and SN. These 14 metabolites were divided into 2 biomarkers and 12 common differential metabolites (CDMs). Upregulation denotes that the content of a metabolite increased in SN when compared with that in SJL. The *Y*-axis is shown as Log^2^FC values for the convenience of visual presentation (Note: The up-amplitude in the text refers to the FC value). The *X*-axis denotes the metabolite name. Abbreviations: 6Hk3OR6OG, 6-hydroxykaempferol-3-*O*-rutinoside-6-*O*-glucoside; kaempferitrin, kaempferol 3,7-dirhamnoside; nicotiflorin, kaempferol 3-*O*-rutinoside; K3OR, kaempferol 3-*O*-robinobioside; K7OR, kaempferol 7-*O*-rhamnoside; kaempferin, kaempferol 3-rhamnoside; astragalin, kaempferol 3-β-d-glucopyranoside; trifolin, kaempferol-3-*O*-galactoside; 6Hk376OT, 6-hydroxykaempferol-3,7,6-*O*-triglycoside; K3OR7OG, kaempferol 3-*O*-rutinoside 7-*O*-glucoside; K7OG, kaempferol 7-*O*-glucoside; 6Hk36OD, 6-hydroxykaempferol-3,6-*O*-diglucoside; Dk, dihydrokaempferol.

Twelves CDMs were classified into kaempferol and kaempferol derivatives. These twelve, as well as two biomarkers identified in this subcategory, are presented here according to the descending order of the FC value from left to right in Figure 6. As shown in Figure 6, these fourteen were all upregulated, indicating that an increase in elevation enhanced the biosynthesis of kaempferol and its derivatives. Furthermore, of these fourteen, 12 are kaempferol glycosides, while only two are aglycones, namely kaempferol and dihydrokaempferol. This further demonstrates that an increase in altitude is beneficial for *Rhodiola crenulata* in terms of accumulating the glycoside form of kaempferol instead of its aglycone form. Moreover, concerning the upregulation amplitude, FC >10 was set as the standard for CDMs with a huge difference, while FC ≤10 was set for CDMs with a relatively low difference. According to this standard, six CDMs with a huge difference were found, shown in Figure 6 on the left, ranging from 6-hydroxykaempferol-3-*O*-rutinoside-6-*O*-glucoside (6Hk3OR6OG) to kaempferin. Their FC values range from 2,081,481.48 to 543.53. All six are kaempferol glucosides that are mainly condensed with rhamnoside, robinobioside, and rutinoside. In comparison, the FC values for the eight remaining on the right range from 8.89–3.22, and all are kaempferol aglycones or glucosides, which are mainly synthesized with glucose. This finding further confirms that an increase in elevation promotes *Rhodiola crenulata* to synthesize kaempferol glycosides, particularly non-glucose forms.

Reports have shown that UV irradiation alters the secondary metabolism of plants, including an enhancement in the production of kaempferol glycosides [34], which exerts a vital protective effect against UV damage [35]. Thus, it is suggested that the buildup of kaempferol and its glucosides may play a powerful role in the resistance of *Rhodiola crenulata* to intensified UV radiation caused by an increase in elevation.

**Figure 7:**
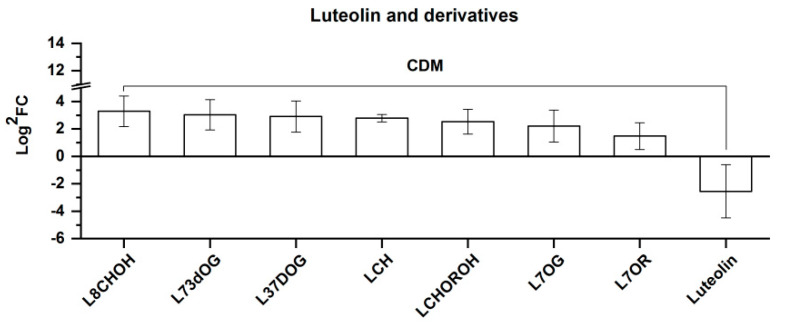
Comparison of eight CDMs of luteolin and its derivatives between SJL and SN. Upregulation denotes that the content of a metabolite increased in SN compared with that in SJL, and the opposite for downregulation. The *Y*-axis is exhibited as Log^2^FC values for the convenience of visual presentation, and the *X*-axis denotes the metabolite names. Abbreviation: L8CHOH, luteolin 8-*C*-hexosyl-*O*-hexoside; L73dOG, luteolin 7,3′-di-*O*-glucuronide; L37DOG, luteolin 3′,7-di-*O*-glucoside; LCH, luteolin *C*-hexoside; LCHOROH, luteolin *C*-hexosyl-*O*-rhamnoside *O*-hexoside; L7OG, luteolin-7-*O*-glucoside; L7OR, luteolin-7-*O*-rutinoside.

Eight CDMs were categorized as luteolin or its derivatives and are displayed according to the order of the FC value from left to right in Figure 7. As shown in Figure 7, all were upregulated with the exception of the rightmost luteolin being downregulated. The characteristics of luteolin and its derivatives were found to be similar to those of kaempferol and its derivatives; however, a difference was found for the downregulation of luteolin, namely the aglycone. This may suggest that a rise in altitude intensifies the biosynthesis of luteolin glycosides in *Rhodiola crenulata* via using luteolin as a potential substrate.

**Figure 8:**
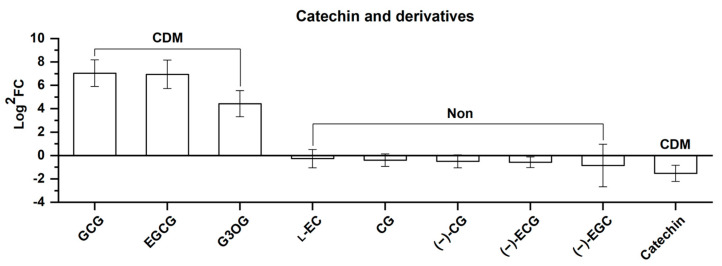
Comparison of nine catechins and catechin derivatives, including 4 CDMs and 5 non-differential metabolites (Non) between SJL and SN. Upregulation denotes that the content of a metabolite increased in SN compared with that in SJL, and the opposite for downregulation. The *Y*-axis is exhibited as Log^2^FC values for the convenience of visual presentation (note that the up and down amplitudes in the text refer to the FC value). The *X*-axis denotes the metabolite names. Abbreviation: GCG, (−)-gallocatechin gallate; EGCG, epigallocatechin gallate; G3OG, gallocatechin 3-*O*-gallate; l-EC, l-epicatechin; CG, catechin gallate; (−)-CG, (−)-catechin gallate; (−)-ECG, (−)-epicatechin gallate; (−)-EGC, (−)-epigallocatechin.

Nine catechins and derivatives were identified, including four CDMs and five non-differential metabolites, shown in Figure 8 in the order of the FC value from left to right. As shown in Figure 8, three upregulated CMDs, namely (−)-gallocatechin gallate (GCG), epigallocatechin gallate (EGCG), and gallocatechin 3-*O*-gallate (131.36, 122.46, and 21.58 fold changes for upregulation, respectively) are catechin derivatives with a relatively complicated structure when compared with the five non-differential metabolites and catechin, i.e., the one downregulated CDM. It should be pointed out that the derivatization degree of catechin is based on the degree of gallate derivatization rather than glycosidation. The results show that elevation influences *Rhodiola crenulata* to produce complex catechin derivatives represented by GCG, EGCG, and gallocatechin 3-*O*-gallate, and that this is highly consistent with the effects of luteolin and derivatives.

Apigenin and its derivatives, quercetin and its derivatives, herbacetin and its derivatives, and hesperetin and its derivatives presented similar features as the above three flavonoids manifested. Nevertheless, exceptions occurred with apigenin and its derivatives, where the upregulated FC value of apigenin was slightly higher than those of apigenin 4-*O*-rhamnoside and apigenin-8-*C*-glucoside (Table S2).

**Figure 9:**
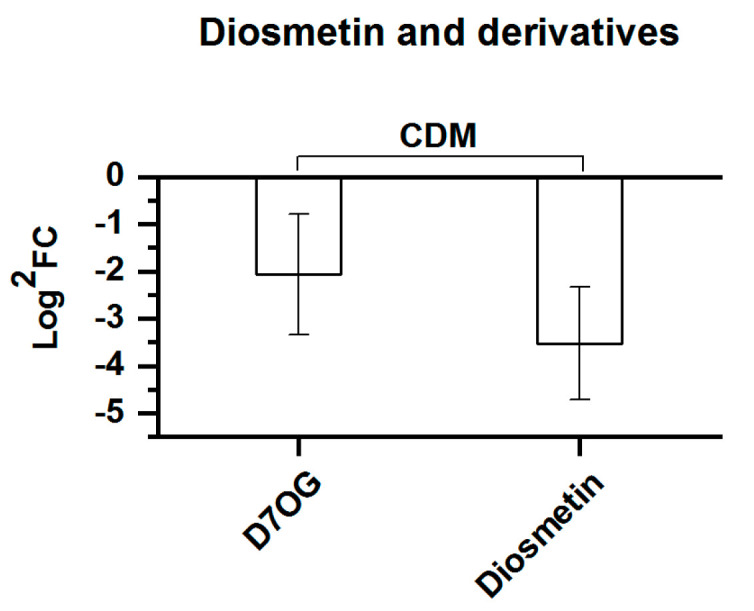
Comparison of diosmetin and its derivatives, comprising 2 CDMs between SJL and SN. Downregulation denotes that the content of a metabolite decreased in SN compared with that in SJL. The *Y*-axis is exhibited as Log^2^FC values for the convenience of visual presentation. The *X*-axis denotes the metabolite names. Abbreviation: D7OG, diosmetin-7-*O*-β-d-glucopyranoside.

The characteristics of diosmetin and its derivatives are partially different from the previously mentioned flavonoids, representing only two CDMs. As shown in Figure 9, a difference was found where the FC values of these two CDMs both declined as the elevation increased. Nevertheless, diosmetin showed a greater decrease in the FC value compared to diosmetin-7-*O*-β-d-glucopyranoside, indicating that *Rhodiola crenulata*, similar to the previously mentioned flavonoids, tends to retain diosmetin glycosides as the altitude rises.

Taken together, the characteristics of flavonoids in *Rhodiola crenulata* when responding to altitude exhibit strong upregulation (recalling 52.69% upregulation versus only 7.53% downregulation of the recognized flavonoids) and a specific inclination to synthesize complex flavonoid glycosides as well as catechin derivatives via gallate derivatization.

Many studies have reported that elevation promotes a notable increase in flavonoids for plants [15,17], and that intensive UV radiation, as well as low temperatures, activate flavonoid glycosylation, leading to the accumulation of flavonoid glycosides in plants, including the stem and root parts [18,19]. Our results are consistent with these previous documents.

Evidence has shown that abiotic stresses cause excessive ROS production in plants, thus triggering oxidative stress [36]. It is known that the antioxidant defense system in alpine plants is more powerful when compared to plants growing at a low altitude [37], and *Rhodiola crenulata* has been validated to possess notable antioxidant activity [38]. Furthermore, flavonoids, as effective antioxidant molecules, display a strong protective effect against cold temperatures and UV radiation in plants [18,39,40]. Research has shown that glycosylation improve the bioactivities of flavonoids, such as an increase in water solubility and the diversification in biological function [41,42], and this is beneficial for plants in terms of tolerating abiotic and biotic stresses [43]. In addition, Huang et al. [44] found that the antioxidant activity exerted by catechins is proportionate to the degree of gallate derivatization.

Based on previous reports, it can be concluded that the upregulation of flavonoids, especially those with a glycoside form, as well as gallate derivatization for catechins, helps *Rhodiola crenulata* endure environmental challenges and presents a strong adaptation to UV radiation, low temperatures, and oxidative stress.

Regarding human beneficial effects, flavonoids are usually restrained by their low bioavailability due to undesirable water solubility, and this defect can be overcome by glycosylation [41,45], thus broadening applications for human health. For instance, hesperetin 7-*O*-glucoside has been reported to be superior to hesperetin and hesperidin for inhibiting human intestinal maltase [46], and the bioactivity of catechins is largely determined by the degree of gallate derivatization [47]. These facts suggest that *Rhodiola crenulata* growing at the higher elevation may possess more advantages for human health applications in the case of flavonoids.

##### Gallic Acid Derivatives

**Figure 10:**
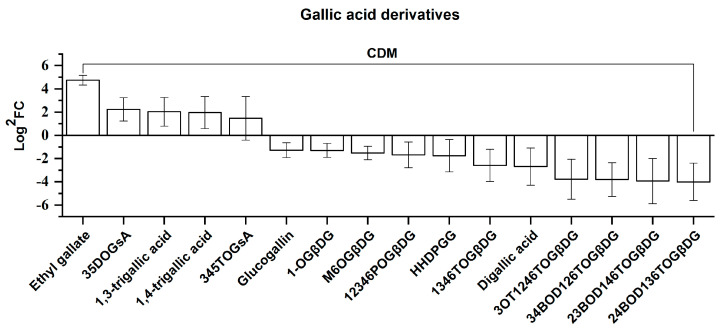
Comparison of gallic acid derivatives, consisting of 16 CDMs between SJL and SN. These 16 CDMs were further divided into 5 upregulated and 11 downregulated CDMs. Upregulation denotes that the content of a metabolite increased in SN compared with that in SJL, and the opposite for downregulation. The *Y*-axis is exhibited as Log^2^FC values for the convenience of visual presentation (note that the up and down amplitudes in the text refer to the FC value). The *X*-axis denotes the metabolite names. Abbreviation: 35DOGsA, 3,5-di-*O*-galloylshikimic acid; 345TOGsA, 3,4,5-tri-*O*-galloylshikimic acid; 1-OGβDG, 1-*O*-galloyl-beta-d-glucose; M6OGβDG, methyl 6-*O*-galloyl-β-d-glucopyranoside; 12346POGβDG, 1,2,3,4,6-penta-*O*-galloyl-beta-d-glucose; HHDPGG, HHDP-galloylglucose; 1346TOGβDG, 1,3,4,6-tetra-*O*-galloyl-beta-d-glucose; 3OT1246TOGβDG, 3-*O*-trigalloyl-1,2,4,6-tetra-*O*-galloyl-beta-d-glucose; 34BOD126TOGβDG, 3,4-bis-*O*-digalloyl-1,2,6-tri-*O*-galloyl-beta-d-glucose; 23BOD146TOGβDG, 2,3-bis-*O*-digalloyl-1,4,6-tri-*O*-galloyl-beta-d-glucose; 24BOD136TOGβDG, 2,4-bis-*O*-digalloyl-1,3,6-tri-*O*-galloyl-beta-d-glucose.

Sixteen CDMs were categorized into this class, containing gallic acid derivatives and hydrolyzed tannins formed by gallic acid and glucose. These 16 CDMs are presented according to the order of their FC value from left to right in Figure 10. As shown in Figure 10, it is apparent that the number of downregulated CDMs is much higher than that of upregulated CDMs (11 downregulated versus 5 upregulated). Furthermore, in terms of the FC values, the 11 downregulated CDM FC values ranged from 0.06–0.41 for the calculation between SN/SJL (also presented as 16.11–2.43 after the conversion of SN/SJL to SJL/SN for the convenience of comparison), which is generally greater than the FC value range for the 4 upregulated CDMs (4.68–2.75), with the exception of 26.64 for ethyl gallate. For the 11 downregulated CDMs, regarding the FC value ranges of five CDMs, namely the CDMs of glucogallin and HHDP-galloylglucose (HHDPGG) and those between them, their values were close to those of the upregulated CDMs, besides ethyl gallate. However, obvious differences from the FC values of the four upregulated CDMs (4.68–2.75) were observed for the remaining six downregulated CDMs displayed on the right, namely those from 1,3,4,6-tetra-*O*-galloyl-beta-d-glucose (1346TOGβDG) to 2,4-bis-*O*-digalloyl-1,3,6-tri-*O*-galloyl-beta-d-glucose (24BOD136TOGβDG).

Regarding the chemical structure, the five upregulated CDMs were comprised of ethyl gallate and two gallic acid polymers (1,3-trigallic acid and 1,4-trigallic acid), including derivatives formed by gallic acid and shikimic acid, namely 3,5-di-*O*-galloylshikimic acid (35DOGsA) and 3,4,5-tri-*O*-galloylshikimic acid (345TOGsA). Regarding the 11 downregulated CDMs, ten were classified into hydrolyzed tannins formed by gallic acid and glucose, except for digallic acid. Among these ten downregulated hydrolyzed tannins, the four presented rightmost in Figure 10 are highly complex in terms of their chemical structures, namely 3-*O*-trigalloyl-1,2,4,6-tetra-*O*-galloyl-beta-D-glucose (3OT1246TOGβDG), 3,4-bis-*O*-digalloyl-1,2,6-tri-*O*-galloyl-beta-d-glucose (34BOD126TOGβDG), 2,3-bis-*O*-digalloyl-1,4,6-tri-*O*-galloyl-beta-d-glucose (23BOD146TOGβDG) and 2,4-bis-*O*-digalloyl-1,3,6-tri-*O*-galloyl-beta-d-glucose (24BOD136TOGβDG), and these exhibited maximum FC values ranging from 0.07 to 0.06. These results demonstrate that hydrolyzed tannins presented a notable downregulation as elevation increased, especially manifesting as structurally complex hydrolyzed tannins.

It is well-accepted that hydrolyzed tannins can protect plants from biotic stress caused by herbivores [20]. Insects display a large distribution at low altitudes, thus imposing serious damage to plants at lower elevations [48]. In addition, there is evidence that UV light leads to a significant degradation of gallic acid in the rhizosphere of plants [49]. Based on previous reports, inferences can be drawn that the downregulation of gallic acid derivatives characterized by hydrolyzed tannins in *Rhodiola crenulata* may be attributed to biotic stress and UV intensity variation with elevation.

##### Phenylpropanoids

**Figure 11:**
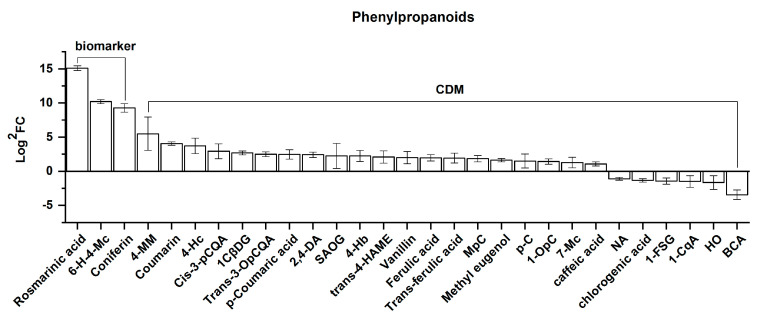
Comparison of phenylpropanoids, comprising 3 biomarkers and 26 CDMs between SJL and SN. Overall, 23 were upregulated and 6 were downregulated. Upregulation represents the content of a metabolite increasing in SN compared with that in SJL, and the opposite for downregulation. The *Y*-axis shows Log^2^FC values for the convenience of visual presentation (note that the up and down amplitudes in the text refer to the FC value). The *X*-axis denotes the metabolite names. Abbreviation: 6-*H*-4-Mc, 6-hydroxy-4-methylcoumarin; 4-MM, 4-methoxycinnamaldehyde; 4-Hc, 4-hydroxycoumarin; *cis*-3-pCQA, *cis*-3-*p*-coumaroyl quinic acid; 1CβDG, 1-caffeoyl-β-d-glucose; *trans*-3-OpCQA, *trans*-3-*O*-*p*-coumaroyl quinic acid; 2,4-DA, 2,4-dihydroxybenzoic acid; SAOG, syringic acid *O*-glucoside; 4-Hb, 4-hydroxybenzaldehyde; *trans*-4-HAME, *trans*-4-hydroxycinnamic Acid methyl ester; MpC, methyl *p*-coumarate; *p*-*C*, *p*-coumaraldehyde; 1-OpC, 1-*O*-*p*-coumaroylglycerol; 7-Mc, 7-methoxycoumarin; NA, neochlorogenic acid; 1-FSG, 1-feruloyl-sn-glycerol; 1-CqA, 1-caffeoylquinic acid; HO, hydrate oxypeucedanin; BCA, brevifolin carboxylic acid.

For the 29 differential phenylpropanoids screened, these contained three biomarkers and 26 CDMs, shown based on the order of the FC value from left to right in Figure 11. As shown in Figure 11, 23 were upregulated while six were downregulated. Moreover, the FC value of the 23 upregulated CDMs ranged from 35,000 to 2.12, specifically 35,000 for rosmarinic acid, 1185.19 for 6-hydroxy-4-methylcoumarin (6-*H*-4-Mc), 629.63 for coniferin, 45.32 for 4-methoxycinnamaldehyde (4-MM), 16.75 for coumarin, 13.29 for 4-hydroxycoumarin (4-Hc), and 7.67–2.12 for the remaining 17, whereas a range of 0.47–0.09 (also expressed as 2.15–10.92 in the form of SJL/SN) for the 6 downregulated CDMs was found. These findings depict an overall upregulation of phenylpropanoids in *Rhodiola crenulata* as elevation increases.

Regarding the chemical structure, it was revealed that these 23 upregulated CDMs are mainly associated with cinnamic acid, coumaric acid, and coumaroyl, presenting either as direct derivatives of these three or as being indirectly developed from these three via the phenylpropanoid biosynthetic pathway, such as caffeic acid, 2,4-dihydroxybenzoic acid, or coniferin [27]. Therefore, based on these findings, a clear pathway can be pictured where the phenylpropanoid pathway, via cinnamic acid, coumaric acid, and coumaroyl-CoA, proceeds towards flavonoid synthesis and that this is enhanced in *Rhodiola crenulata* as elevation increases. Combined with the findings from the gallic acid derivatives, it is clearly presented that elevation causes a conversion of the gallic acid pathway, forming hydrolyzed tannins, to the phenylpropanoid pathway, leading to flavonoid production in *Rhodiola crenulata*.

It has been documented that low temperatures and UV radiation activate the plant flavonoid biosynthetic pathway via the activation of PAL and chalcone synthase, which are the two rate-limiting enzymes at the beginning in the phenylpropanoid and flavonoid biosynthetic pathways, respectively [27,50,51,52,53,54]. Therefore, it can be inferred that enhancement of the phenylpropanoid pathway, proceeding to flavonoid production in *Rhodiola crenulata*, is highly related to the UV intensity and temperature variation with elevation. In addition, rosmarinic acid and coniferin, the two biomarkers, also exhibit an effective protective effect against UV damage [55,56].

##### Amino Acids

**Figure 12:**
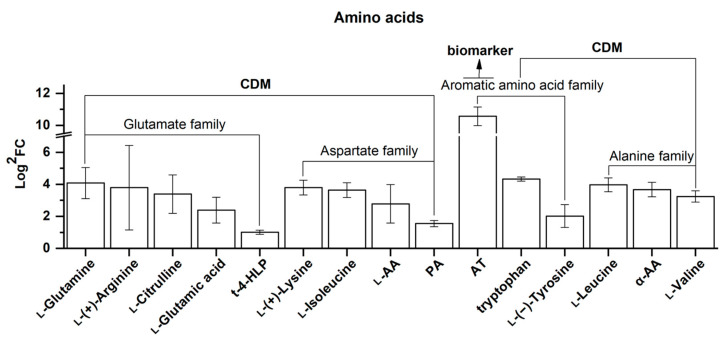
Comparison of amino acids, consisting of 1 biomarker and 14 CDMs between SJL and SN. They are clustered into four families, namely the glutamate family, aspartate family, aromatic amino acid family, and alanine family. Upregulation denotes that the content of a metabolite increased in SN compared with that in SJL. The *Y*-axis is exhibited as Log^2^FC values for the convenience of visual presentation (note that the amplitude in the text refers to the FC value). The *X*-axis denotes the metabolite names. Abbreviation: *t*-4-HLP, *trans*-4-hydroxy-l-proline; l-AA, l-asparagine anhydrous; PA, pipecolic acid; AT, acetyl tryptophan; α-AA, alpha-aminocaproic acid.

Of the 15 differential amino acids screened, all were upregulated, including 1 biomarker and 14 CDMs (Figure 12). As shown in Figure 12, they are clustered into four amino acid families, namely, the glutamate family, aspartate family, aromatic amino acid family, and alanine family, and these are shown in the order of the FC value from left to right in Figure 12.

In the glutamate family, five upregulated CDMs are presented here, ranging from l-glutamine (16.97 for the FC value) to *trans*-4-hydroxy-l-proline (2.01 for the FC value). It is well recognized that glutamine and glutamic acid (also known as glutamate) are essential substrates for aminotransferases to produce various amino acids in the amino acid biosynthetic pathway [57], and thus their upregulation may suggest that amino acid biosynthesis is enhanced in *Rhodiola crenulata* as elevation is increased, which is in accordance with the results where all the differential amino acids screened here were upregulated. Moreover, a report has shown that arginine promotes root development [58]. Additionally, hydroxyproline, as the main composition of hydroxyproline-rich glycoprotein (HRGP) in the cell wall, is closely related to the formation of cambium, phloem parenchyma, and various types of sclerenchyma [59]. Thus, its upregulation may be a sign denoting the normal growth of *Rhodiola crenulata* as elevation is increased.

Considering the aspartate family, four upregulated CDMs are shown here, ranging from l-(+)-lysine (13.90 for the FC value) to pipecolic acid (2.93 for the FC value). l-asparagine is well-known to be located at the beginning of the aspartate biosynthetic pathway, thus its upregulation may be indicative of activation of the aspartate biosynthetic pathway in *Rhodiola crenulata,* which is consistent with upregulation of l-(+)-lysine and l-isoleucine, which are the downstream products of this pathway. Reports have shown that lysine helps improve plant drought tolerance [25] and isoleucine is closely related to plant resistance to salt stress [26] and the growth of germinating embryos [60].

Of the three aromatic amino acids, all were upregulated, including acetyl tryptophan with a FC value of 1524.07, tryptophan with 20.09, and l-(−)-tyrosine with 4.06. Tryptophan is well-known as the key precursor for tryptophan-dependent pathways of IAA biosynthesis in plants [61,62]. Tyrosine may help improve plant drought tolerance [63]. In addition, phenylalanine, as a non-differential metabolite, also presented an upregulation of 1.65 for the FC value, suggesting that it might be, as a beginning substrate [27], directly consumed in phenylpropanoid-flavonoid biosynthesis.

Taken together, these upregulated amino acids may be responsible for the adaption of *Rhodiola crenulata* to environmental challenges caused by elevation via the strengthening drought and salt tolerances, and additionally phenylpropanoid-flavonoid biosynthesis. Possibilities also exist regarding the promotion of root development, auxin synthesis, and cell wall biosynthesis via the effects of these amino acids.

##### Alkaloids

**Figure 13:**
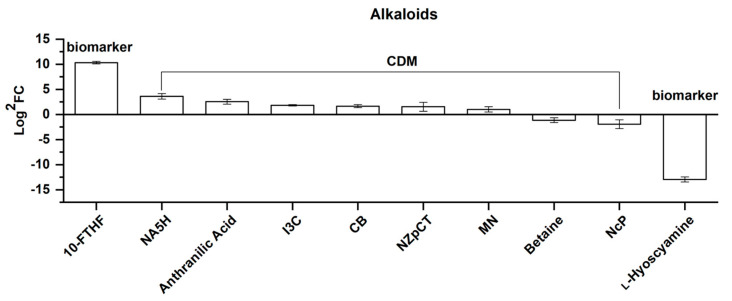
Comparison of alkaloids, consisting of 2 biomarkers and 8 CDMs between SJL and SN. Upregulation denotes that the content of a metabolite increased in SN compared with that in SJL, and the opposite for downregulation. The *Y*-axis is exhibited as Log^2^FC values for the convenience of visual presentation. The *X*-axis denotes the metabolite names. Abbreviation: 10-FTHF, 10-formyl-THF; NA5H, *N*-acetyl-5-hydroxytryptamine; I3C, indole-3-carboxaldehyde; CB, cocamidopropyl betaine; NZpCT, *N*-*Z*-*p*-coumaroyl-tyramine; MN, methyl nicotinate; NcP, *N*-*cis*-paprazine.

Of the 10 differential alkaloids screened, seven were upregulated and three were downregulated (Figure 13). Figure 13 shows these alkaloids in the order of their FC value from left to right, and they consist of two biomarkers and eight CDMs.

UV intensity and low temperatures, which are the environmental factors relating to elevation, have been reported to impose alterations to plant alkaloid accumulation [22,23,64], therefore providing an insight into our findings. Research has shown that the formation of *N*-acetyl-5-hydroxytryptamine and indole-3-carboxaldehyde is related to auxin biosynthesis [65,66,67]. Our results show that that *N*-acetyl-5-hydroxytryptamine and indole-3-carbaldehyde were both upregulated here. Thus, considering the strong upregulations of both acetyl tryptophan and tryptophan, a hypothesis can be proposed where the tryptophan-dependent pathways of IAA biosynthesis are enhanced in *Rhodiola crenulata* as the growth elevation is increased. However, indole-3-acetic acid has not been detected in our data. Thus, this hypothesis requires further validation. In addition, methyl nicotinate, a vasodilator, has been reported to cause an acceleration of blood circulation [68]. The upregulation of methyl nicotinate here may provide a new clue about study on the anti-hypoxic effect exerted by *Rhodiola* [69].

##### Organic Acids

**Figure 14:**
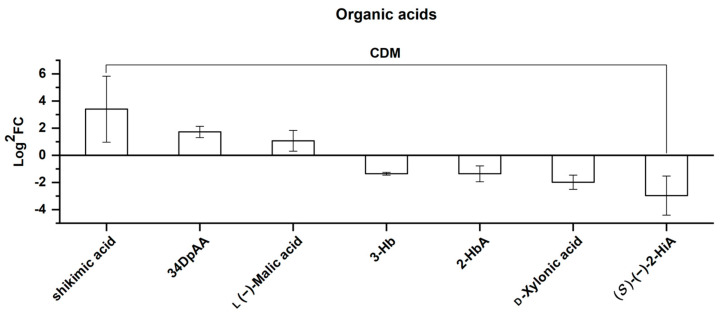
Comparison of organic acids, consisting of 7 CDMs between SJL and SN. Upregulation denotes that the content of a metabolite increased in SN compared with that in SJL, and the opposite for downregulation. The *Y*-axis is exhibited as Log^2^FC values for the convenience of visual presentation. The *X*-axis denotes the metabolite names. Abbreviation: 34DpAA, 3,4-dimethoxyphenyl acetic acid; 3-Hb, 3-hydroxybutyrate; 2-HbA, 2-hydroxybutanoic acid; (*S*)-(−)-2-HiA, (*S*)-(−)-2-hydroxyisocaproic acid.

Of the seven differential organic acids screened here, three were upregulated and four were downregulated (Figure 14). As shown in Figure 14, in the order of the FC value from left to right, all were CDMs.

Many organic acids are involved in the plant citric acid cycle. Three non-differential metabolites, namely citric acid, succinic acid, and fumaric acid, as well as the upregulated malic acid, were identified here, being indicative of a good survival of *Rhodiola crenulata* at a high altitude, presented by the existence of organic acids involved in the citric acid cycle [24]. Malic acid is a vital index for indicating the occurrence of crassulacean acid metabolization [70]. Thus, its upregulation may suggest that an increase in crassulacean acid metabolization occurs in *Rhodiola crenulata* (belonging to *Sedum*) as elevation increases. The shikimic acid pathway, highlighted by shikimic acid, converts simple carbohydrate precursors derived from glycolysis and the pentose phosphate pathway to aromatic amino acids, further proceeding to the phenylpropanoid and flavonoid biosynthetic pathways [27]. Thus, the upregulation of shikimic acid, combined with the enhancement of the downstream phenylpropanoid-flavonoid pathway previously discovered, jointly depicts an approximate whole picture of the secondary metabolism of *Rhodiola crenulata* as elevation is increased.

## 3. Materials and Methods 

### 3.1. Plant Material and Extract Preparation

Four *Rhodiola* samples were included in this study: SJL (collection location: Sejila Mountain region, Tibet, China; altitude of 2907 m; 94°54′35.96′′E, 29°59′16.83′′N), SN (collection location: Shannan, Tibet, China; altitude of 5116 m; 91°37′23.34′′E, 29°23′3.99′′N), JL1 (collection location: Changbai Mountain, Jilin, China; altitude of 1950 m; 128°1′19.68′′E, 41°58′30.41′′N) and JL2 (collection location: Changbai Mountain, Jilin, China; altitude of 2248m; 128°0′49.48′′E, 42°1′10.5′′N). For each *Rhodiola* sample, three wild-grown *Rhodiola* plants were collected as biological replicates during September of 2018. SJL and SN were authenticated as *Rhodiola crenulata* (J. D. Hooker & Thomson) H. Ohba and JL1 and JL2 were authenticated as *Rhodiola rosea* Linnaeus by Dr. Shubin Dong at the Beijing Forestry University. After freeze-drying, rhizomes of the collected samples were ground using a mill, then sieved through a #40 mesh to obtain a dried powder. Subsequently, the powders were stored at −20 °C.

To prepare extracts, 15 mL of 50% methanol was added to 1.5 g of the dried powder and the mixture was sonicated in a water bath for 30 min at room temperature, then filtrated through a 0.22 μm filter to collect the supernatant. This extraction process was repeated twice more. Afterwards, these three supernatants were pooled together and stored at 4 °C in preparation for the process outlined in Section 3.2.

### 3.2. Determinations of Phenolic Components and Antioxidant Abilities

The total phenols, total flavonoids, total tannins, and DPPH and ABTS antioxidant abilities were measured via following the previous reports [71,72]. All assays were conducted in triplicate.

### 3.3. UPLC-QqQ-MS-Based Metabolomics

The *Rhodiola* samples (SJL and SN) were prepared by freeze-drying and milled with a zirconia bead, and a QC sample was obtained by mixing SJL and SN in order to measure the assay’s reproducibility. Subsequently, 100 mg of each sample was extracted overnight at 4 °C with 0.6 mL of 70% aqueous methanol. Following centrifugation at 10,000× *g* for 10 min, the extracts were absorbed by a Carbon-GCB SPE Cartridge (CNWBOND Carbon-GCB SPE Cartridge, 250 mg, 3 mL; ANPEL, Shanghai, China, www.anpel.com.cn/cnw) and then filtrated through a 0.22-μm membrane (SCAA-104, 0.22-μm pore size; ANPEL, Shanghai, China, www.anpel.com.cn) to obtain the filtrate.

The filtrates were analyzed using a LC-ESI-MS/MS system (HPLC, Shim-pack UFLC SHIMADZU CBM30A system, SHIMADZU, Kyoto, Japan; MS, Applied Biosystems 4500 Q TRAP, Applied Biosystems, Foster City, CA, USA). The analytical conditions were as follows: A Waters ACQUITY UPLC HSS T3 C18 (1.8 µm, 2.1 mm × 100 mm, Waters, Milford, MA, USA) was used as the column; the mobile phase consisted of solvent A: Pure water with 0.04% acetic acid, and solvent B: Acetonitrile with 0.04% acetic acid. Measurements were conducted with a gradient program as follows: 0 min: 95% A, 5% B; 0–10 min: A linear gradient to 5% A, 95% B; 10–11 min: 5% A plus 95% B was kept; a combination of 95% A with 5.0% B was set within 0.10 min and kept running for 11.1–13.0 min. The column oven was set to 40 °C and the injection volume was 4 μL. The effluent was connected to an ESI-triple quadrupole-linear ion trap (QqQ-LIT)-MS.

Linear ion trap (LIT) and triple quadrupole (QqQ) scans were obtained by a triple quadrupole-linear ion trap mass spectrometer (API 4500 Q TRAP LC/MS/MS System, AB Sciex, Framingham, MA, USA, http://www.absciex.com/) equipped with an ESI Turbo Ion-Spray interface. This was operated in the positive and negative ion modes and controlled by the Analyst 1.6.3 software package (AB Sciex, Framingham, MA, USA, http://www.absciex.com/). The ESI source operation parameters were the following: Ion source: Turbospray; source temperature: 550 °C; ion spray voltage: 5500 V; ion source gas I, gas II, and curtain gas (CUR) were set at 50, 60, and 25.0 psi, respectively; the collision gas (CAD) was high. Instrument tuning and mass calibration were conducted with 10 and 100 μmol/L polypropylene glycol solutions in the QqQ and LIT modes, respectively. The QqQ scans were acquired by MRM assays with a collision gas (nitrogen) setting of 5 psi. The declustering potential (DP) and collision energy (CE) measurements for individual MRM transitions were carried out with further DP and CE optimizations. A specific set of MRM transitions were monitored for each period according to the metabolites eluted within this period.

For metabolite qualitative determination, primary and secondary mass-spectrometry data were analyzed based on the MetWare database (MWDB), which is a self-built database. MWDB was also reported in previous studies [73,74].

### 3.4. Statistical Analysis

Multivariate statistical analysis was performed using the R software package (Bell Laboratories, Inc. (formerly AT&T, now Lucent Technologies) by John Chambers and colleagues, Kinsman BLVD, Madison, WI, USA, www.r-project.org) according to the report of [73], including principal component analysis (PCA), orthogonal partial least squares discriminant analysis (OPLS-DA), hierarchical clustering analysis (HCA), and heatmap construction. The overall data were first subjected to PCA to transform the original variables into a few principal components as a new complex index through orthogonal transformations. The data were also subjected to HCA to create a heatmap. Based on the OPLS-DA results, the variable importance in project (VIP) values and fold changes were used to screen for differential metabolites between samples with the criteria of simultaneously meeting a VIP value ≥1 and FC ≥2 or ≤0.5. Biomarkers were identified by the high abundance of certain metabolites in a *Rhodiola* sample (i.e., SN) versus zero abundance (set by 9.00 cps) of the corresponding metabolite in another *Rhodiola* sample (i.e., SJL).

## 4. Conclusions

The *Rhodiola* species is a traditional alpine medicine in Asian and European countries and exerts various pharmacological effects. The influence of different *Rhodiola* species on quality and the influence of altitude on its phytochemicals demands prompt investigation. In the present study, two major *Rhodiola* species, namely *Rhodiola crenulata* and *Rhodiola rosea*, were compared. Systemic changes in the phytochemicals of *Rhodiola crenulata* from two representative elevations of 2907 and 5116 m were characterized here.

Our findings here can be summarized as follows: (1) the phenolic components and antioxidant activities in *Rhodiola crenulata* are greater than those in *Rhodiola rosea*, and these two species both present a positive correlation with elevation regarding the phenolic components and antioxidant activities. This indicates that *Rhodiola crenulata* possesses better quality and that altitude is an influencing factor for phytochemicals. (2) Here, 19 biomarkers for differentiating *Rhodiola crenulata* in two different elevations were screened. (3) Here, the significant upregulation of flavonoids featuring glucosides, the enhancement of the phenylpropanoid pathway, and the downregulation of hydrolyzed tannins were characterized by increase elevation in *Rhodiola crenulata*. (4) The amino acids of all samples displayed upregulation as altitude increased. Our findings provide new insights into the phytochemical response of *Rhodiola crenulata* to elevation. *Rhodiola crenulata* grown at a higher elevation could be explored as a good source of phytochemicals for health benefits related to *Rhodiola*.

## Figures and Tables

**Table 1:** Biomarkers for *Rhodiola crenulata* from different altitudes.

Metabolite	Category	Fold Change	Type
6-Hydroxykaempferol-3-*O*-rutinoside-6-*O*-glucoside	Flavonoids	2,081,481.48	Up
Cyanidin 3-*O*-glucoside	Flavonoids	358,518.52	Up
Rosmarinic acid	Phenylpropanoids	35,000.00	Up
Tricetin	Flavonoids	11,303.70	Up
Engeletin	Flavonoids	8877.78	Up
Turanose sodium salt	Others	6618.52	Up
Apigenin 7-*O*-glucoside (cosmosiin)	Flavonoids	5022.22	Up
Kaempferol 3,7-dirhamnoside (kaempferitrin)	Flavonoids	4618.52	Up
3-Hydroxyhippuric acid	Others	3025.93	Up
Hexadecyl ethanolamine	Others	1646.30	Up
Acetyl tryptophan	Amino Acid	1524.07	Up
10-Formyl-THF	Alkaloids	1275.19	Up
6-Hydroxy-4-methylcoumarin	Phenylpropanoids	1185.19	Up
9-β-d-Arabinofuranosylhypoxanthine	Nucleotides	1080.00	Up
LysoPE 18:1 (2n isomer)	Lipids	923.33	Up
Coniferin	Phenylpropanoids	629.63	Up
l-Hyoscyamine	Alkaloids	0.0001	Down
5,7-Dihydroxychromone	Others	0.00001	Down
Quillaic acid	Others	0.000003	Down

Note: 1. Upregulation denotes that the content of a metabolite in SN exhibited a significant increase compared to that in SJL. Conversely, downregulation indicates the content of a metabolite in SN is significantly decreased compared with the corresponding one in SJL; 2. Biomarkers were identified by the high abundance of certain metabolites in a *Rhodiola* sample (i.e., SN) versus zero abundance (set by 9.00 cps) of the corresponding metabolite in another *Rhodiola* sample (i.e., SJL); 3. Biomarkers are presented according to the descending order of the fold change value from top to bottom.

## Supplementary Materials

The following are available online, Table S1: List of identified metabolites between quality control, SJL, and SN; Table S2: List of differential metabolites between SJL and SN; Table S3: List of biomarkers between SJL and SN; Figure S1: Model verification of OPLS-DA.
