# Supplementary material for: Evaluation of Two Major Rhodiola Species and the Systemic Changing Characteristics of Metabolites of Rhodiola crenulata in Different Altitudes by Chemical Methods Combined with UPLC-QqQ-MS-Based Metabolomics
Source: Molecules. 2020 Sep 5;25(18):4062. doi: 10.3390/molecules25184062 (PMC7570721; doi:10.3390/molecules25184062)
Supplement: Supplementary file 1 [file molecules-25-04062-s001.zip › molecules-910473-SM/Figure S1.docx]

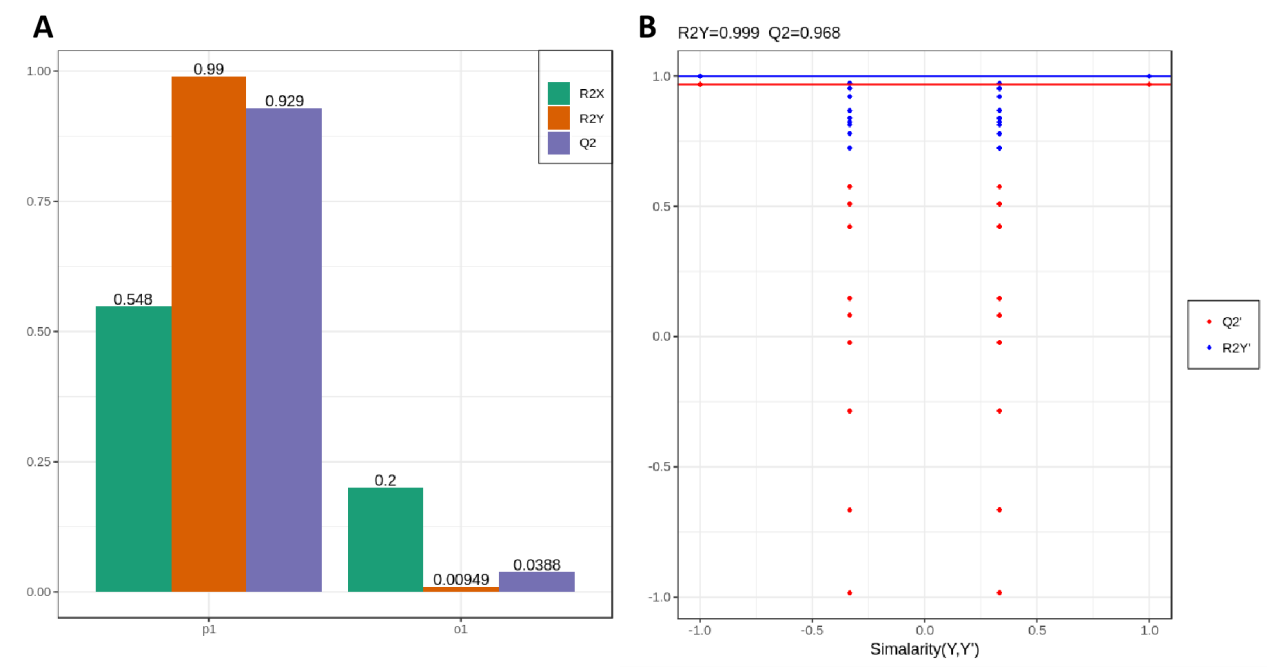


**Figure S1.** Model verification of OPLS-DA. A: OPLS-DA model profiles for SJL vs SN. B: permutation test of the OPLS-DA model of SJL vs SN. High predictability (Q2) and strong goodness of fit (R2X, R2Y) of the OPLS-DA models were presented by SJL vs SN (Q2 = 0.929, R2X = 0.548, R2Y = 0.99; Figure S1A). In figure S1B, the horizontal lines correspond to R2Y and Q2 of the original model, and the red and blue dots denote R2Y' and Q2' of the model following Y replacement. R2Y' and Q2' are both smaller than R2Y and Q2 of the original mode exhibited by location below the corresponding lines, indicating that this model are credible and differential metabolites screened by VIP value analysis are valid.
